# Percutaneous consolidation of bone metastases: strategies and techniques

**DOI:** 10.1186/s13244-019-0709-7

**Published:** 2019-02-06

**Authors:** Roberto Luigi Cazzato, Julien Garnon, Benham Shaygi, Emanuele Boatta, Guillaume Koch, Jean Palussiere, Xavier Buy, Afshin Gangi

**Affiliations:** 1 Department of Interventional Radiology, Nouvel Hôpital Civil (Hôpitaux Universitaires de Strasbourg), 1 Place de l’Hôpital, 67000 Strasbourg, France; 2 Department of Interventional Radiology, Royal Devon and Exeter Hospital NHS Trust, Barrack Rd, Exeter, EX2 5DW UK; 3 Department of Interventional Radiology, Institut Bergonié, 229 cours de l’Argonne, 33000 Bordeaux, France

**Keywords:** Bone, Metastases, Fractures, Osteoplasty, Osteosynthesis

## Abstract

Patients with cancer can present with bone metastases (BM), which are frequently complicated by different types of fractures necessitating prompt management to avoid serious impairment in terms of quality of life and survival.

Percutaneous image-guided bone consolidation has rapidly emerged as an alternative to surgical fixation and is mainly reserved for patients who are deemed unfit for surgical management. Two percutaneous techniques, osteoplasty and osteosynthesis, are available and are selected based on the biomechanics of the target bones as well as the fracture types.

The aim of this narrative review is to present the different types of BM-related fractures and the interventional strategies and techniques underpinning their minimally invasive percutaneous fixation.

## Keypoints

- Bone metastases are frequently complicated by three different types of fractures.
- Percutaneous image-guided osteoplasty and osteosynthesis can be used to fix cancer-related bone fractures.
- Percutaneous osteoplasty and osteosynthesis should be mainly offered to “non-surgical” patients.

## Introduction

Bone metastases (BM) represent a common clinical condition in cancer patients as bone-metastasizing tumors such as prostate, breast, and lung cancer account for approximately 45% of cancers [1, 2]. Clinically, BM often result in pain, fractures, and hypercalcemia [2]; moreover, surgery or radiation therapy (RT) is frequently required to manage these presentations. These events are commonly known as skeletal-related events (SREs). Amongst SREs, fractures represent one of the most troublesome complications as they can cause significant pain, functional disability, and neurological sequelae, dramatically affecting quality of life and survival. Surgical fixation has traditionally been the treatment of choice due to its construct durability. However, it is frequently deemed unsuitable for frail oncology patients generally due to perioperative factors such as prolonged anesthetic time and prolonged recovery time, adding again to a risk of reduction in quality of life and survival [3]. For this reason, the minimally invasive, percutaneous image-guided techniques which are associated with a shorter recovery time have been introduced with encouraging results [4–13].

The aim of this narrative review is to present the different types of BM-related fractures and the interventional strategies and techniques underpinning their percutaneous fixation.

### Type of fractures

**Fig. 1:**
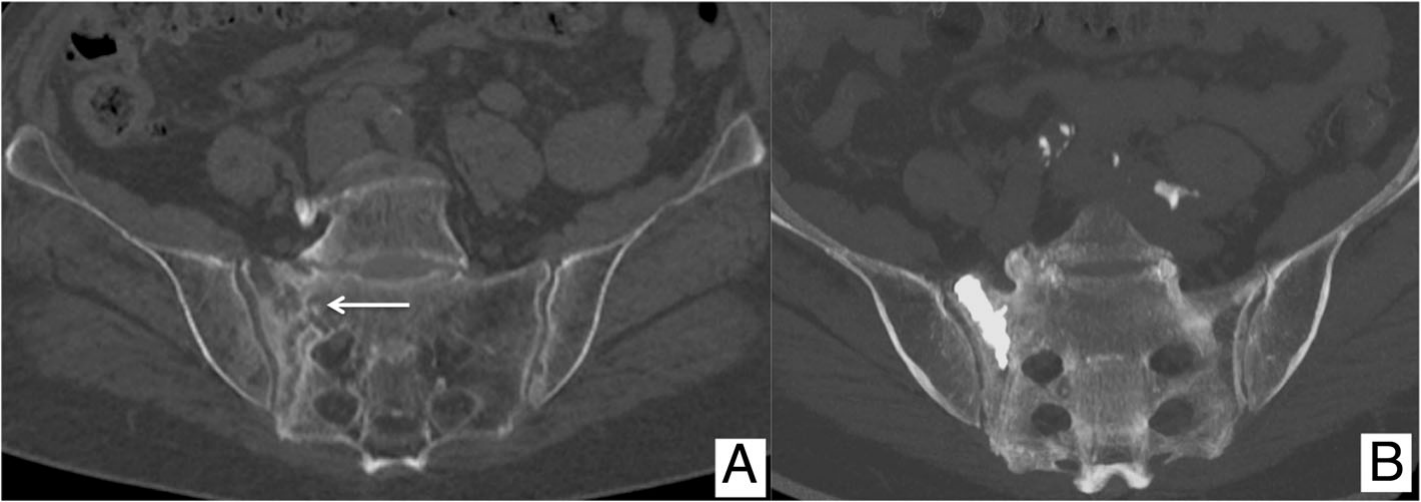
Eighty-four-year-old female patient with breast cancer, presenting with (**a**) a painful insufficiency fracture of the right sacral wing (arrow). **b** Coronal MIP CT image demonstrating the result of the sacroplasty

**Fig. 2:**
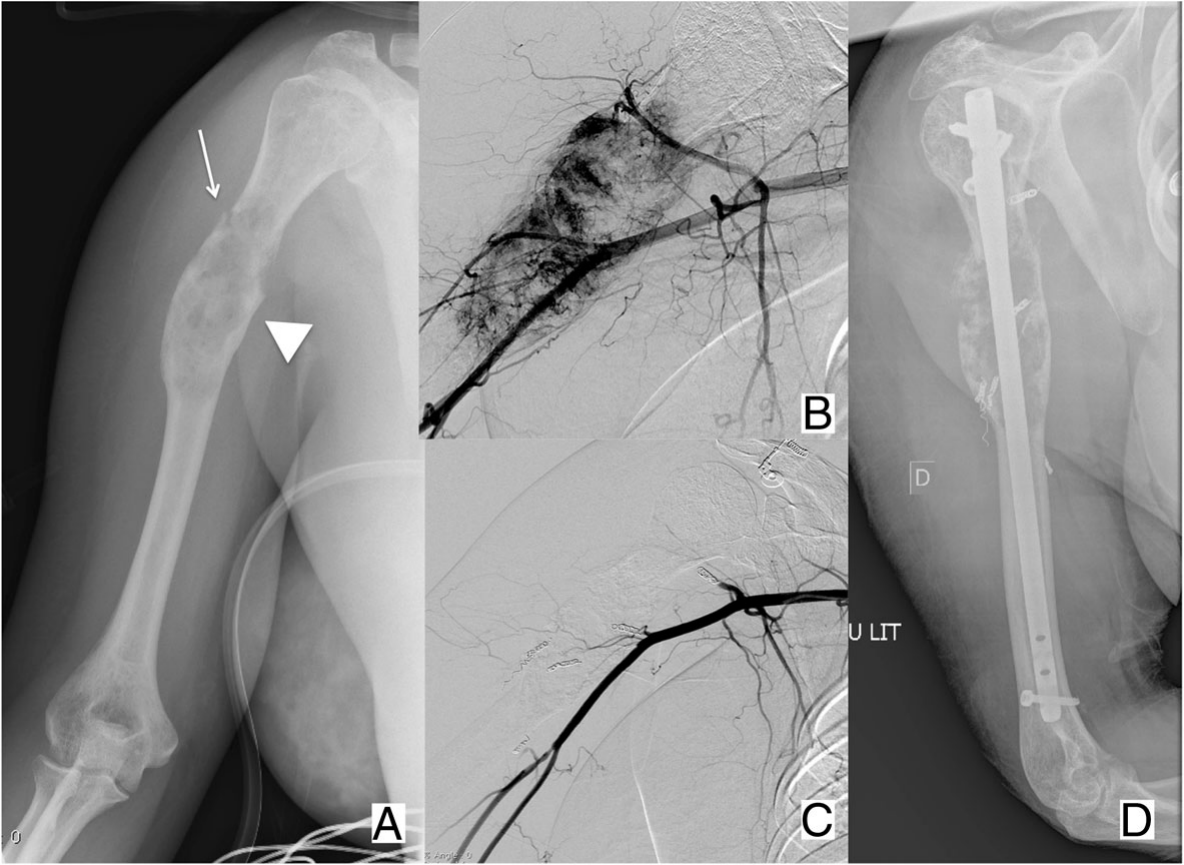
Forty-six-year-old female patient with kidney cancer, presenting with (**a**) a painful metastasis of the diaphysis of the right humerus (arrow head), complicated by a non-displaced pathologic fracture (arrow). **b**, **c** Given the hyper-vascular nature of the metastasis, embolization was performed before (**d**) surgical fixation to limit the risk of intra-operative bleeding

**Fig. 3:**
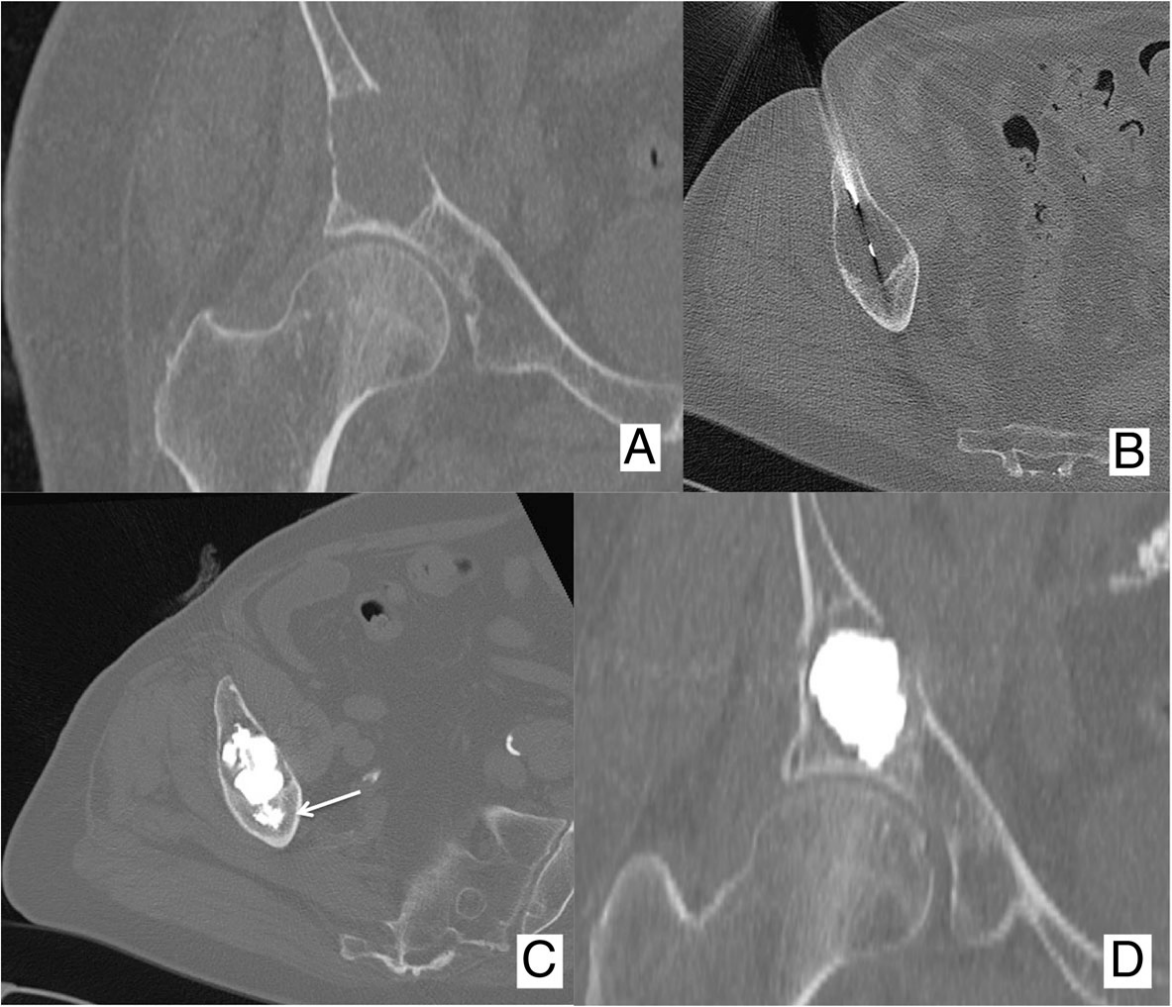
Eighty-five-year-old male patient presenting with an acute mechanic pain of the right hip. **a** A CT scan revealed a large lytic lesion of the acetabulum without any sign of pathologic fracture. The patient underwent (**b**) percutaneous biopsy that revealed a metastasis from kidney cancer; **c**, **d** in the same session, the patient received osteoplasty with fast and effective pain relief. Of note, PMMA was anchored in the distal normal bone (arrow) before filling the lytic cavity

Oncology patients may present with three different types of fractures [13]:

- *Bone insufficiency fractures* resulting from the bone necrosis secondary to percutaneous ablation or radiotherapy or resulting from the bone resorption as a result of tumor metabolism or certain treatments (e.g., chemotherapy, long-term steroid treatment) (Fig. 1).
- *Pathologic fractures* resulting from bone replacement by infiltrating tumor (Fig. 2).
- *Impending fractures* being consistent with painful and extensive metastatic tumor involvement of the weight-bearing bones, which are therefore at an increased risk of fracture; subsequently, preventive consolidation is highly advised (Fig. 3).

### Interventional strategies

Percutaneous bone consolidation is strictly applied to “non-surgical” cancer patients. This includes those patients being unsuitable for surgical management due to the suboptimal physiological state, refusal of consent, or unacceptable delay to systemic therapy. These patients are treated, provided they have an acceptable estimated life expectancy (> 1 month) [9, 13].

Percutaneous consolidation can be performed as a stand-alone interventional procedure having the sole purpose of the fracture fixation or as part of a more complex strategy, which combines percutaneous consolidation with the ablative therapy within the same interventional session. The latter alternative is generally reserved for the patients presenting with an impending or pathologic fracture:

- Requiring focal treatment to achieve local tumor control due to their oligometastatic (< 3–5 metastases, each < 3 cm) or oligoprogressing (1 to 3 metastases evolving despite good systemic tumor control assured by systemic therapies) status [14–17].
- Demonstrating soft-tissue infiltration requiring tumor debulking to prevent the complications to the adjacent organs or to control pain [15].

Contraindications to percutaneous bone consolidation are as follows: severely displaced fractures, concurrent osteomyelitis or active systemic infection, severe uncorrectable coagulopathy, and allergy to the bone cement or osteosynthesis material.

### Percutaneous techniques and their selection

#### Osteoplasty

**Fig. 4:**
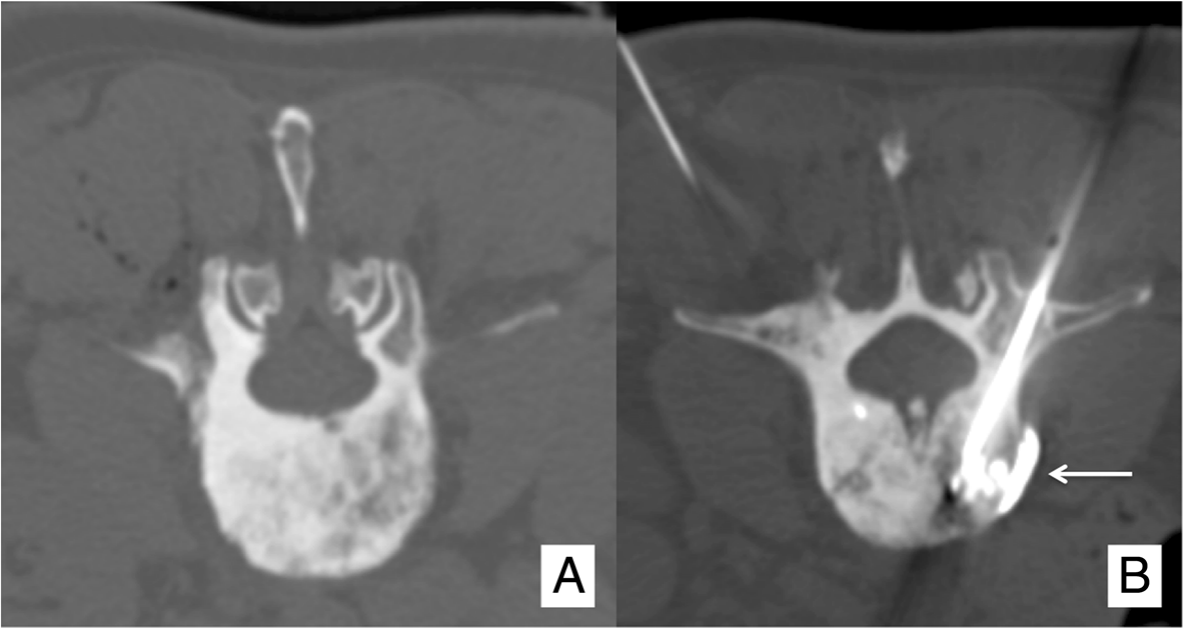
Vertebroplasty performed in a (**a**) sclerotic vertebral metastasis. **b** The amount of PMMA injected was very limited, and an early non-symptomatic para-vertebral leakage occurred (arrow)

The basic principle of osteoplasty is to fill a bone cavity or a fractured bone with poly-methyl-methacrylate (PMMA; Figs. 1 and 3). Osteoplasty should not be applied to treat sclerotic BM (Fig. 4). Osteoplasty is applied in bones where compressive stress is predominant [18]. In bones where torsion, bending or shearing stresses occur, osteoplasty should not be applied since PMMA is not resistant to these mechanic solicitations. Although osteoplasty prevents compression fracture, a secondary fracture may still occur especially in case of huge local tumor progression.

In order to inject the PMMA, a safe and stable bone access should be gained under CT or fluoroscopic guidance. The bone access is often achieved by the means of a 10–13 G bevelled bone trocar, which is manually hammered in the target bone so that its distal tip is safely anchored in the normal distal bone. Then, the liquid and solid compositions of the PMMA (Table 1) are mixed together for few minutes until toothpaste-like consistency is achieved. Injection is performed within 15–20 min, before PMMA polymerization occurs. The polymerization phase results in an exothermic reaction with transient but significant (up to 75 °C) temperature rise, which is however not adequate to induce complete and effective tumor necrosis since the tumoricidal effect is limited to 3 mm around the PMMA [7, 19].

**Table 1:** Polymethylmetacrylate features

Solid phase composition	• PMMA pre-polymer and/or copolymers of acrylic acid (AA) • Activator of the polymerization: benzoyl peroxide • Radiopacifiers: barium sulfate, zirconium dioxide, tantalum, and tungsten
Liquid phase composition	• Methyl methacrylate monomer • Activator of the polymerization: *N*-*N*-dimethyl-p-toluidine (DMPT) • Inhibitor of polymerization during storage: hydroquinone (HQ)
Bending modulus Bending strength Compressive strength	• 2600–3500 MPa • 46–76 MPa • 70–111 MPa

PMMA is injected through a dedicated gun-like device, under continuous fluoroscopic guidance to monitor PMMA distribution within the target bone and to detect as early as possible any potential PMMA leakage. To avoid irradiation to operators’ hands, leaded gloves should be used. The injection is commenced in the distal normal bone in order to anchor the PMMA in healthy bone; thereafter, the trocar tip is gently withdrawn and the injection is continued to fill the lytic cavity as much as possible (Fig. 3). In the case of any PMMA leakages outside the bone, the injection should be immediately stopped especially if vascular leakages are noted. Disruption of the normal cortical bone does not represent an absolute contraindication to osteoplasty [8, 20] even though there is a theoretical increased risk of PMMA leakage. In the end, it should be noted that although PMMA leakage is the most common adverse event, it rarely results into a clinically significant complication [8].

#### Osteosynthesis

**Fig. 5:**
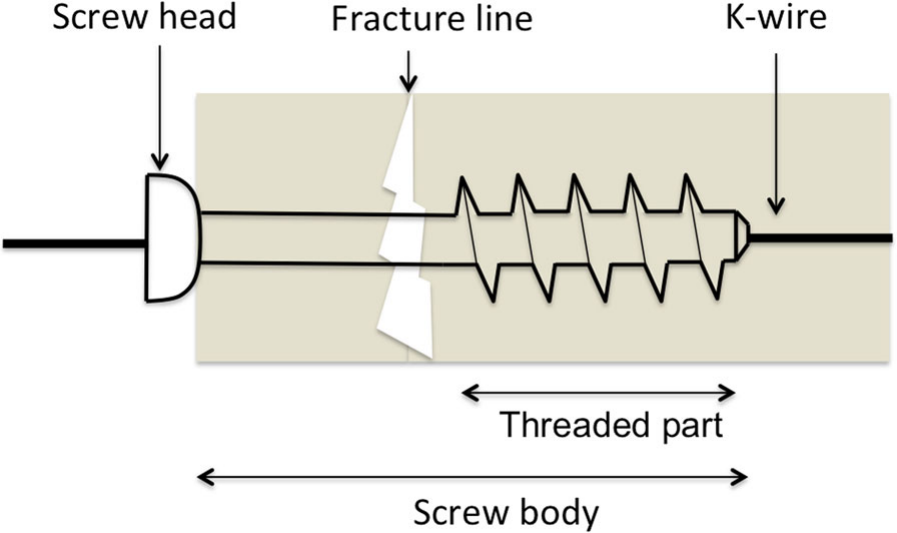
Screw components and deployment. k-wire: Kirschner wire

**Fig. 6:**
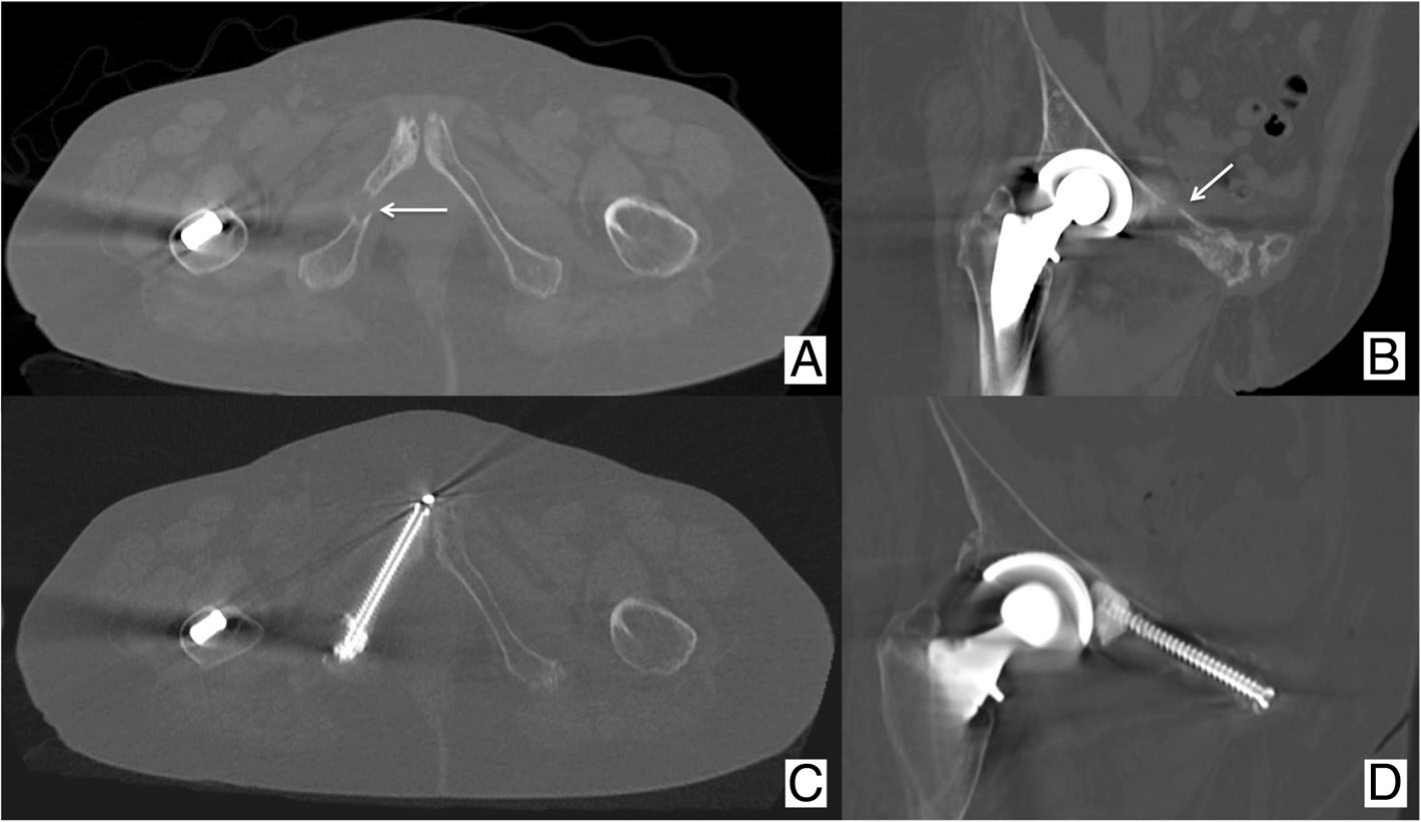
Same patient as Fig. 1 presenting also with painful pathologic fractures (arrows) of the right (**a**, **b**) ischio-pubic and ilio-pubic ramus. **c**, **d** Both fractures were fixed percutaneously with the cannulated PMMA-injectable screws

**Fig. 7:**
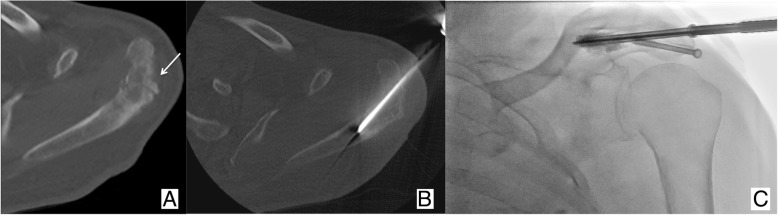
Seventy-four-year-old male patient affected by lung cancer. The patient underwent radiation therapy of a painful metastasis of the left acromion, which was complicated few weeks later by (**a**) a secondary bone insufficiency fracture. **b**, **c** Percutaneous osteosynthesis was proposed to fix the fracture under combined CT and fluoroscopy guidance; two screws were deployed with subsequent rapid pain relief

Osteosynthesis aims to bridge the fracture line or the lytic BM with 6–7.2-mm cannulated threaded screws that convert rotational forces into linear motion (Fig. 5). Percutaneous osteosynthesis can be applied to fix fractures and lytic BM mainly located within the pelvic ring (Fig. 6) and occasionally in the shoulder girdle (Fig. 7) [5, 11, 12].

The screws are made of stainless steel or titanium, comprised of a head and a body, and are self-drilling/self-tapping to avoid the jamming of the cut bone whilst being advanced into the target bone.

Screws are manually advanced by means of a dedicated screwdriver over a 1.8–2-mm Kirschner wire, which is deployed in the target bone coaxially through a 10G bone trocar either directly into the bone by means of an electric drill.

Kirschner wire deployment represents the most critical phase of the procedure, thus often requiring expert operators and advanced imaging guidance such as combined CT/fluoroscopy (Fig. 7) or cone-beam CT.

The screws need to be anchored in proximal and distal healthy bone by perpendicularly bridging the fracture line. They also ought to bridge the lytic BM parallel to the long axis of the target bone, in order to allow maximal inter-fragmentary compression. In particular, the head of the screw should abut the external cortical bone of the proximal bone fragment, and the distal part of the body should be anchored in the distal healthy bone (Fig. 5). Before deploying the screws, a dedicated calliper is used coaxially over the Kirschner wire in order to select the most adapted screw length.

Screws with the partially threaded part located only at the distal aspect of the body allow the best inter-fragmentary compression and are therefore indicated for minimally displaced fractures. Screws with a fully threaded body are indicated for non-displaced fractures. PMMA-injectable screws (Fig. 6) provided with multiple holes at the distal part of the body are indicated whenever there is a need to increase the screw anchoring within the distal bone such as in severely osteoporotic patients.

Secondary fractures are unlikely following osteosynthesis unless massive local tumor progression occurs. Nevertheless, unfavorable local evolution consistent with poor consolidation of the treated site or screw loosening has been described in up to 12.5% cases at mean 8.7-month follow-up with up to 1/3 patients being symptomatic [13]. Therefore, clinic/imaging follow-up is warranted following osteosynthesis.

### Selection of the consolidative technique

Whilst making a choice between the osteoplasty and osteosynthesis, the predominant biomechanics of the target bone as well as the type of fracture should be taken into account.

#### Spine

In the spine, vertebroplasty is highly effective in consolidating the insufficiency fractures or the painful lytic BM involving the vertebral bodies. Tumor infiltrations into the posterior wall of the vertebral body or the anterior epidural space, without significant spinal cord compression, do not contraindicate vertebroplasty [20], provided that the operators are highly experienced with the procedure and high-quality fluoroscopy is available. In cases of vertebral instability (which has been shown to be accurately calculated by the means of the “Spine Instability Neoplastic Score” (SINS) [21]) resulting from extensive tumoral involvement of the posterior vertebral elements, vertebroplasty is contraindicated and surgical approach is warranted. Surgical referral for urgent decompressive laminectomy should also be considered in all cases presenting with the emerging neurological symptoms related to the severe neoplastic compression of the spinal cord.

#### Pelvic area

Painful lytic supra-acetabular BM represent a suitable indication for percutaneous osteoplasty in this area of high compressive stress [22]. Nevertheless, if the acetabular BM is complicated by a fracture, osteosynthesis should be considered [5, 13]. Osteosynthesis is also indicated in cases of minimally or non-displaced fractures of the iliac wing or the ilio/ischio-pubic ramus as well as the midline fractures of the sacrum [5, 13]. In the end, if sacral wings fractures are noted, osteoplasty is indicated [23].

#### Long bones

Consolidation is warranted by a Mirels’ score ≥ 8 (Table 2) [24]. Nevertheless, percutaneous techniques are of limited use here and are reserved for a very few selected patients. In particular, given the significant inadaptability of PMMA to support stresses such as torsion or bending, osteoplasty alone results in a relatively high risk (8–9%) of secondary fractures [6, 8]. Accordingly, long bone osteoplasty is only applied for strictly non-surgical patients presenting with small lytic epiphyseal BM without articular involvement and very limited life expectancy. Particular care should be taken to avoid intra-articular PMMA leakage, since it may result in deleterious complications (i.e., rapidly evolving chondrolysis) [25].

**Table 2:** Mirels’ score: A score ≥ 8 indicates prophylactic consolidation

	1 point	2 points	3 points
Lesion Aspect	Blastic	Mixed	Lytic
Cortical Involvement	< 1/3	1/3–2/3	> 2/3
Site	Upper limb	Lower limb	Trochanteric region
Pain	+/−	Moderate	Mechanic

**Fig. 8:**
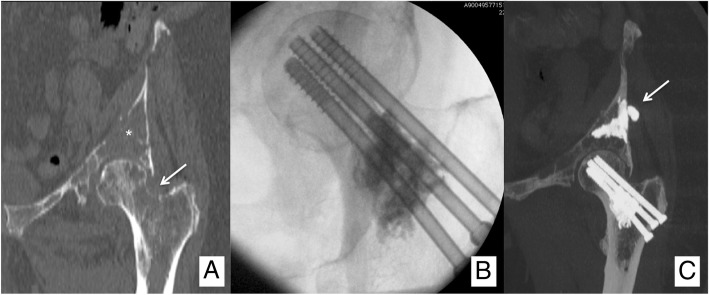
Sixty-three-year-old female patient affected by breast cancer, presenting with (**a**) painful lytic metastases in the acetabulum (*) and in the proximal femur (Mirels’ score: 10; arrow). **b** The patient received an osteosynthesis of the femoral neck with an inverted triangle configuration coupled to PMMA injection to fill the lytic cavity. **c** In the same session, percutaneous osteoplasty of the acetabulum was performed, and a small asymptomatic PMMA leakage in the nearby soft tissues was noted (arrow)

Osteosynthesis, however, can be applied in femoral neck fractures with an inversed triangular configuration (Fig. 8), provided that they are minimally or non-displaced and there is no massive cortical disruption or involvement of the trochanteric region [9]. Finally, diaphyseal or metaphyseal fractures warrant surgical fixation in almost all the cases. Nevertheless, new minimally invasive percutaneous techniques based on the insertion of metallic wires into the medullary cavity have been sporadically reported in “non-surgical” cancer patients [26, 27] and are likely to become available to interventional radiologists in the next future, provided that larger series will corroborate the long-term efficacy.

## Conclusions

Several different types of bone fractures are encountered in cancer patients. The percutaneous image-guided fixation approaches can be considered and offered to “non-surgical” patients after careful evaluation of the predominant biomechanics of the target bones as well as the type of fractures.

## Abbreviations

BM: Bone metastases
PMMA: Poly-methyl-methacrylate
RT: Radiation therapy
SINS: “Spine Instability Neoplastic Score”
SREs: Skeletal related events
